# Supplementary material for: Perception and awareness of COVID-19 among health science students and staff of Kuwait University: An online cross-sectional study
Source: F1000Res. 2021 Jul 14;10:566. [Version 1] doi: 10.12688/f1000research.54046.1 (PMC11167330; doi:10.12688/f1000research.54046.1)
Supplement: Peer review history

# Reviewer report: Reviewer response for version 1

**DOI:** 10.5256/f1000research.57491.r281362

**Published:** 2024-06-11

**Authors:** Mahmoud Abdel Hameed Shahin

**Affiliations:** 1 Prince Sultan Military College of Health Sciences, Dammam, Saudi Arabia

**Review of "Perception and awareness of COVID-19 among health science students and staff of Kuwait University: An online cross-sectional study "**

**Strengths of the Research Methodology and Process:**

 The study used a cross-sectional online survey design appropriate for assessing the perception and awareness of COVID-19 among the target population at a specific time.

 The questionnaire was divided into six relevant sections covering socio-demographics, risk and awareness, preparedness and prevention, sources of information, policies, and social stigma. This comprehensive approach allowed for a thorough assessment of various aspects related to COVID-19.

 The study included both students and staff of the health science departments at Kuwait University, providing a broader perspective on the topic.

 The researchers took steps to ensure the study's tool validity, including questionnaire validation and expert reviewers' involvement.

 Generally, the study provides valuable insights into the perception and awareness of COVID-19 among Kuwait University health science students and staff. However, the research methodology and process could be further strengthened to enhance the generalizability of the findings.

**Areas for Improvement:**

 You mentioned “HCS” in the methods of the abstract, which needs to be expressed in full for the first time.

 In the data collection tool, you discussed the questionnaire's validity and piloting, but nothing was mentioned about the tool's reliability testing. It is recommended that the test-retest reliability testing or Cronbach’s alpha coefficient testing be mentioned.

 After the conclusions, it is advised to add the recommendations of the study, which comprise the essence of the study findings for the readers to implement and utilize.

 Adding the limitations of the study gives more transparency and guides other researchers in terms of research chances of improvement.

Is the work clearly and accurately presented and does it cite the current literature?

Yes

If applicable, is the statistical analysis and its interpretation appropriate?

Yes

Are all the source data underlying the results available to ensure full reproducibility?

Yes

Is the study design appropriate and is the work technically sound?

Yes

Are the conclusions drawn adequately supported by the results?

Yes

Are sufficient details of methods and analysis provided to allow replication by others?

Yes

Reviewer Expertise:

Critical care, COVID-19, Medical Education, Nursing, Enteral Nutrition.

I confirm that I have read this submission and believe that I have an appropriate level of expertise to confirm that it is of an acceptable scientific standard.

## References

[1] : Risk perception regarding the COVID-19 outbreak among the general population: a comparative Middle East survey. *Middle East Current Psychiatry* .2020;27(1) : 10.1186/s43045-020-00080-7 10.1186/s43045-020-00080-7

# Reviewer report: Reviewer response for version 1

**DOI:** 10.5256/f1000research.57491.r99758

**Published:** 2021-11-29

**Authors:** Moawiah M. Hussein Khatatbeh

**Affiliations:** 1 Department of Basic Medical Sciences, Faculty of Medicine, Yarmouk University, Irbid, Jordan

This is an interesting article about awareness of COVID-19 among health science students and staff of Kuwait University. The comparisons between students and staff are unique in relation to COVID-19 literature.

 Figure 1 presents "Participants awareness of consequences of COVID-19 patients". I assume these are attitudes not awareness. Moreover, I would prefer a 3-point Likert Scale. Participants can express their attitudes towards a particular item more easily.

Is the work clearly and accurately presented and does it cite the current literature?

Yes

If applicable, is the statistical analysis and its interpretation appropriate?

Yes

Are all the source data underlying the results available to ensure full reproducibility?

Yes

Is the study design appropriate and is the work technically sound?

Yes

Are the conclusions drawn adequately supported by the results?

Yes

Are sufficient details of methods and analysis provided to allow replication by others?

Yes

Reviewer Expertise:

Public Health research

I confirm that I have read this submission and believe that I have an appropriate level of expertise to confirm that it is of an acceptable scientific standard.
